# Privacy-Constrained Biometric System for Non-Cooperative Users

**DOI:** 10.3390/e21111033

**Published:** 2019-10-24

**Authors:** Mohammad N. S. Jahromi, Pau Buch-Cardona, Egils Avots, Kamal Nasrollahi, Sergio Escalera, Thomas B. Moeslund, Gholamreza Anbarjafari

**Affiliations:** 1 Visual Analysis of People Laboratory, Aalborg University, 9100 Aalborg, Denmark; kn@create.aau.dk (K.N.); tbm@create.aau.dk (T.B.M.); 2 Computer Vision Centre, Universitat Autònoma de Barcelona, 08193 Bellaterra (Cerdanyola), Barcelona, Spain; pbuch@cvc.uab.es (P.B.-C.); sergio@maia.ub.es (S.E.); 3 iCV Lab, Institute of Technology, University of Tartu, 50411 Tartu, Estonia; ea@icv.tuit.ut.ee (E.A.); shb@icv.tuit.ut.ee (G.A.); 4 Department of Mathematics and Informatics, Universitat de Barcelona, 08007 Barcelona, Spain; 5 Department of Electrical and Electronic Engineering, Hasan Kalyoncu University, 27900 Gaziantep, Turkey

**Keywords:** biometric recognition, multimodal-based human identification, privacy, deep learning

## Abstract

With the consolidation of the new data protection regulation paradigm for each individual within the European Union (EU), major biometric technologies are now confronted with many concerns related to user privacy in biometric deployments. When individual biometrics are disclosed, the sensitive information about his/her personal data such as financial or health are at high risk of being misused or compromised. This issue can be escalated considerably over scenarios of non-cooperative users, such as elderly people residing in care homes, with their inability to interact conveniently and securely with the biometric system. The primary goal of this study is to design a novel database to investigate the problem of automatic people recognition under privacy constraints. To do so, the collected data-set contains the subject’s hand and foot traits and excludes the face biometrics of individuals in order to protect their privacy. We carried out extensive simulations using different baseline methods, including deep learning. Simulation results show that, with the spatial features extracted from the subject sequence in both individual hand or foot videos, state-of-the-art deep models provide promising recognition performance.

## 1. Introduction

Biometric recognition is the science of identification of individuals based on their biological and behavioral traits [1,2]. In the design of a biometrics-based recognition or authentication system, different issues, heavily related to the specific application, must be taken into account. According to the literature, ideally biometrics should be universal, unique, permanent, collectable, and acceptable. In addition, besides the choice of the biometrics to employ, many other issues must be considered in the design stage. The system accuracy, the computational speed, and cost are important design parameters, especially for those systems intended for large populations [3]. Recently, biometric recognition systems have posed new challenges related to personal data protection (e.g., GDPR), which is not often considered by conventional recognition methods [4]. If biometric data are captured or stolen, they may be replicated and misused. In addition, the use of biometrics data may reveal sensitive information about a person’s personality and health, which can be stored, processed, and distributed without the user’s consent [5]. In fact, GDPR has a distinct category of personal data protection that defines ‘biometric data’, its privacy, and legal grounds of its processing. According to GDPR, what qualifies as ‘Biometric data’ is defined as ‘personal data resulting from specific technical processing relating to the physical, physiological or behavioural characteristics of a natural person, which allow or confirm the unique identification of that natural person such as facial images’ [6]. Furthermore, GDPR attempts to address privacy matters by the preventing of processing any ‘sensitive’ data revealing information such as health or sexual orientation of individuals. In other words, processing of such sensitive data can be only allowed if it falls under ten exceptions laid down in GDPR [6]. Apart from this privacy concern, in some scenarios, designing and deploying a typical biometric system where any subject has to cooperate and interact with the mechanism may not be practical. In care homes with elderly patients, for example, interaction of the user with typical device-dependent hardware or following specific instruction during biometric scan (e.g., direct contact with a camera, placing a biometric into a specific position, etc.) [7,8]. In other words, the nature of such uncontrolled environments suggest the biometric designer to consider strictly natural and transparent systems that mitigate the user non-cooperativeness behavior, providing an enhanced performance.

This possibility was explored in our earlier work [9] by considering identification of persons when they grab the door handle, which is an unchanged routine, in opening a door and no further user training is required. In our previous work, we designed a bimodal dataset (hand’s dorsal, hereafter refer to as hand, and face) by placing two cameras above the door handle and frame, respectively. This was done in order to capture the dorsal hand image of each user while opening the door for multiple times (10 times per user) in a nearly voluntary manner. In addition, face images of users approaching the physical door were collected as a complementary biometric feature. In [9], we concluded that facial images are not always clearly visible due to the nonoperative nature of the environment, but, when visible, it provides complementary features to hand-based identification.

In [9], however, the study disregards the privacy of the users previously mentioned here as all the methods employ the visible face of each subject in the recognition task, which is considered as sensitive information in the new data protection paradigm.

In this paper, we deal with the problem of automatic people recognition under privacy constraints. Due to this constraint, it is crucial to conduct a careful data-collection protocol that excludes any sensitive biometric information that may comprise user’s privacy. For instance, to protect the users, acquiring facial or full-body gait information of candidates is not possible. Consequently, we have collected a new data-set containing only the hands and feet of each subject using both RGB and near/infrared cameras. We verified the usefulness of the designed setup for user privacy-constrained classification by performing extensive experiments with both conventional handcrafted methods as well as recent Deep Learning models.

The remainder of this paper is organized as follows: Section 2 discusses related work in the field. In Section 3, the database is presented. In Section 4, the dataset is evaluated with classical and deep learning strategies. Finally, conclusions are drawn in Section 5.

## 2. Related Work

This section reviews the existing methods on hand and the footprint recognition focusing mostly on the use of geometric spatial information. There are a few detailed studies that are reviewing different hand-based biometric recognition systems [10,11]. Visual specifications of hands constitute a paramount criterion for biometric-based identification of persons, owing to the associated respectively low computational requirements and mild memory usages [12]. In addition, they provide superior distinctive representations of persons, which lead to unparalleled recognition success rates. Furthermore, the related procedures can be well adapted into the existing biometric authentication systems, which make them favorable for the foregoing purpose [13,14,15,16,17]. These systems, depending on the type of the features they extract from the hand, can be categorized as follows:

- Group 1: in which the geometric features of the hand are used for the identification. Examples of such features include the length and the width of the hand palm. Conventional methods such as General Regression Neural Network (GRN) [18], graph theory [18], or later methods like sparse learning [19] are examples of this group.
- Group 2: in which hand vein patterns are used for the identification. These patterns are unique to every individual and are not affected by aging, scars and skin color [20]. Therefore, the vascular patterns of an individual’s hand (palm, dorsal or finger) can be used as a feature for biometric recognition systems. An example of this category includes wavelet and Local binary patterns (LBP) based [21,22] or recent deep learning-based methods [23]. Such features have been used in connection with CNNs [24] and extracted using thermal imaging and Hausdorff distance based matching [20,25], and using multi-resolution filtering [26].
- Group 3: in which palm prints are used for identification. Palm prints can be extracted according to texture, appearance, orientations or lines. Besides various conventional techniques, there are dictionary and deep learning methods [27,28] reported in literature. Considering the above categories, the geometry-based hand features are robust to both rotation and translation. However, at the same time, they are not suitable to scale variations. Moreover, in order to achieve high performance for the recognition task, a huge amount of measurements is needed to extract discriminative features of each subject. This will eventually increase the computational complexity. The hand vein features, on the other hand, are robust to varying hand poses and deformation. They may also introduce computational cost if all distances between landmark points are required. Finally, for the palm-print based recognition, some methods that achieve high recognition rates exist, but, in general, acquiring high-resolution palm-print images is challenging due to setup complexities.

### Footprint

Contrary to many well-established biometric techniques used in the context of automatic human recognition, the human foot features are rarely used as a feature in those solutions. Although the uniqueness property of the human foot is extensively addressed in the forensic studies [29], its commercial solution is considered mostly complicated due to complexity of the data acquisition in the environment [30]. The very early attempt of employing a human foot as means of identification emerged in the forensic study carried out by Kennedy [29] in which he examines the uniqueness of barefoot impression. In [31], the first notion of utilizing the Euclidean distance between a pair of human feet was presented. In [32], the authors propose a static and dynamic footprint-based recognition based on a hidden Markov model. The latter implemented a footprint based biometric system, similar to a hand, which involves exploiting the following foot features:

- Group 1: in which the shape and geometrical information of the human foot are used for identification. Features of this category concentrate on the length, shape and area of the silhouette curve, local foot widths, lengths of toes, eigenfeet features and angles of intertoe valleys [30]. The research works in [33,34,35,35] are a few examples of this category. In general, a variety of possible features makes shape and geometric-based methods very popular. In addition, these methods are robust to various environmental conditions. The drawback of such a large number of possible features, however, can eventually result in high intrapersonal variability.
- Group 2: in which the texture-based information of the human foot are used for identification. In this group, pressure (soleprint features analogous to palm print based of the hand biometric) and generated heat can be considered as the promising features. Examples in this category can be found in [30,36]. Unlike the shape and geometrical features of feet, acquiring a fine-grained texture of feet requires a high accuracy instrument. For example, skin-texture on palm-print involves extracting rather invisible line patterns as opposed to the similar one in the hands. Similar challenges may exist in recording ridge structure with high resolution. On the other hand, the high-resolution of texture-based features will require higher computational power with respect to shape and geometrical ones.

Minutiae-based ballprint [30] in the foot as well as different distance techniques such as city-block, cosine, and correlation [37] are further examples of the features that are employed in this context. It is also important to mention that gait biometrics [38] are also a potential approach that studies the characteristic of human foot strike.

## 3. Acquisition Setup

In this paper, in order to have a realistic testing environment, an acquisition setup has been designed by employing a standard-size building door with three camera sensors, one mounted above its handle, and two installed at the frame side, respectively.

**Figure 1:**
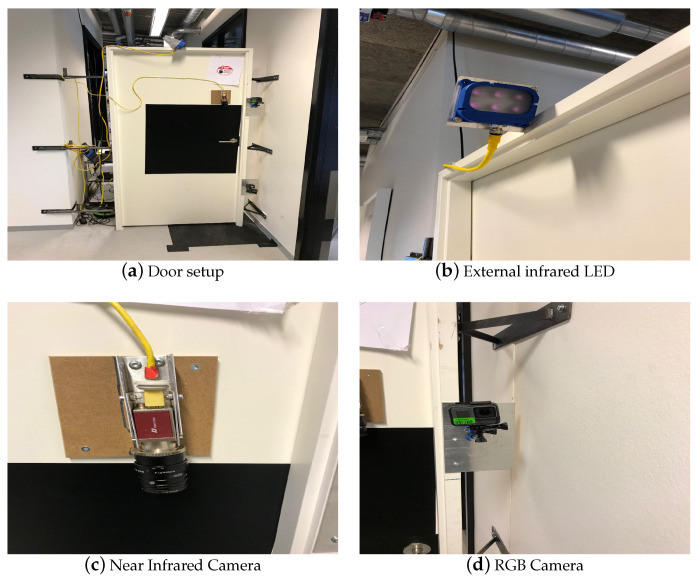
Illustration of the main set-up and the sensors.

During data collection, it is important to capture each modal in a clear visible form so that all unique meaningful features can be extracted. In other words, each modal has to be collected by a proper sensor. In this work, for example, each subject approaches a door and grabs its handle to open it. Therefore, each subject’s hand should be recorded by a sensor while placed on the door handle. Based on several conducted tests with different available sensors, we choose to employ a near infrared light (NIR) camera (AV MAKO G-223B NIR POE) equipped with a band pass filter to cut off visible light. In this way, for hands, good feature candidates such as veins can be properly extracted. In addition, to guarantee that the hand modals on the door handles are visible in the captured frames, a near infrared light source (SVL BRICK LIGHT S75-850) was also mounted on the door frame. To capture each foot modal, a regular RGB camera (GoPro Hero 3 Black) on the door frame is installed to capture the subject’s foot as they approach the door. The third camera in this setup has been used to acquire the face modality of each corresponding subject although it is not used to perform automatic classification. They are collected to conduct alternative studies beyond the scope of this paper and hence excluded. The overall door model together with the installed cameras and the light source are shown in Figure 1.

**Figure 2:**
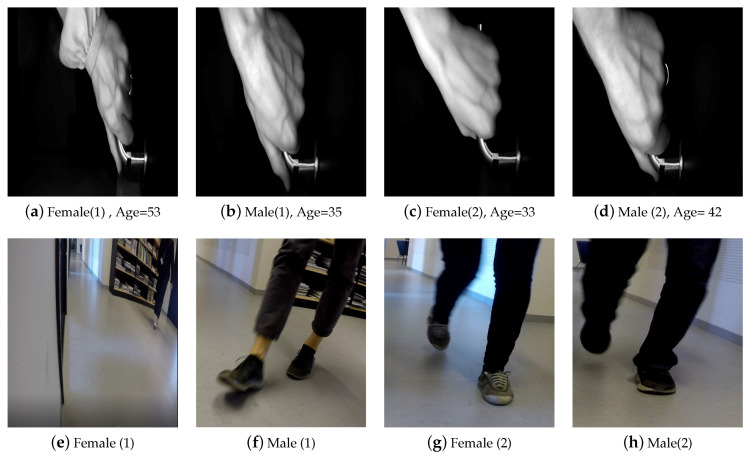
Sample of captured frames of both hand and foot modalities for four subjects.

A total of 77 persons of mixed gender and varying ages from 20 to 55 years participated in the data collection procedure at *Aalborg University*. There exist three paths that each subject can take to approach the door setup. Each person is requested to approach the door from any desired path randomly. These paths can cover both linear and curvature trajectory, making the scenario natural. The participant then walks toward to the door, grabs its handle and then passes through the door. This procedure is repeated two times. During data acquisition, no further instructions were given to the participants. This is done to have the participants grab the door handle as they would naturally perform in any context. As a result, all data are captured in a totally natural scenario where a variety of realistic situations such as occlusion, different pose and partial foot may occur. Furthermore, the lighting condition is not controlled and the data has been collected during different times of day for two months. Figure 2 shows samples acquired by the different cameras.

Each video sequence of the subject’s hand/foot is post processed to enhance the quality of captured frames and remove any camera distortion. This is performed by using the well-known chessboard camera calibration tool in the vision library of MATLAB (2019, MathWorks) [39].

Privacy disclaimer: While our proposal moves in the direction of privacy constrained scenarios, we are aware that still some soft biometrics features used in this work could be used in some situations by specific external observers that could be able to identify the user. Without loss of generality, we use privacy-constrained to refer to the scenario where sensitive user information is avoided, making the biometric identification harder in case data are leaked.

## 4. Experimental Results and Discussion

In this section, we first discuss the evaluation protocol of the experiments. Then, we briefly explain the methods used and finally the obtained results and discussions.

### 4.1. Evaluation Protocol

In order to carry out experiments using different methods, we divided the database into mutually disjoint subsets of training, validation, and testing. As there are two cycles of complete action per modality (i.e., each user approaches the door twice), each video sample is divided into two sequences per modality. Next, we use all first sequences from all subjects for both hands and feet to train while utilizing the second sequence of the subjects for validation and testing, respectively. In this manner, we have 77 sample sequences per modality for training, 37 sequences for validation and 40 samples for the test. Each test sample is then associated with a label during simulations.

In this paper, the main focus of all the experiments is around general spatial appearance models. In other words, for all the simulations, the spatial features are extracted through the analysis of each independent frame (uncorrelated frames per same subject). For the evaluated deep learning model, we have further analyzed the contribution of the motion as an input modality. Finally, we have also performed late-fusion on both modalities for all of the experiments.

To summarize, the performed experiments are divided into the following three categories:

- ***Independent frame analysis***: The final evaluation is based on frames independently. That is, frames from the same subject are uncorrelated. The maximum output probability from each frame determines its final predicted class.
- ***Subject sequence analysis***: The final evaluation is based on grouping frames from the same sequence/subject. We average output probabilities belonging to the same subject, and we finally obtain the maximum output value as its final predicted class.
- ***Hand–Feet Late Fusion Analysis***: Averaged output probabilities from hand and feet outputs (aka subject sequence analysis) are averaged together for each subject to finally determine the final predicted class.

### 4.2. Conventional Techniques Evaluation

#### 4.2.1. Local Binary Patterns and Support Vector Machine

Even tough deep neural networks dominate state-of-the-art solutions in image processing, it is still worthwhile to further test conventional methods to create baseline results, in particular in scenarios where a limited amount of annotated data are available. Local binary patterns (LBP) [40,41,42] are one of the most powerful handcrafted texture descriptors. The core implementation and its variants are extensively used in facial image analysis, including tasks as diverse as face detection, face recognition and facial expression analysis. Benzaoui et al. [43] showed that classification tasks which use LBP for feature extraction can improve various statistical procedures, such as principal component analysis (PCA) and discrete wavelet transform (DWT). For example, by using a combination of DWT, LBP and support vector machine (SVM) [44,45,46] for classification, it is possible to create a hybrid method for face recognition. Similarly, the same approach can be used for hand and foot classification. The performance of an LBP based feature extractor can be greatly improved, making input data robust against certain image transformations. For example, in the case of face images, this relates to aligned and cropped faces. When considering recordings of human gait, the size and foot orientation is constantly changing, thus adding additional challenges to the description and classification problems. The DWT method is widely used in feature extraction, compression and denoising applications. The process of recognition using DWT is as follows: the wavelet transform of a particular level is applied on the test image and the output is an approximation coefficient matrix, which we consider as a sub-image. Then, we extract rotation invariant LBP feature vectors from the sub-images for SVM training and classification [47]. The regions of interest from the video sequences are extracted using the frame difference method across multiple frames. This approach was robust enough to successfully pre-process all the videos in the database. The system was developed in MATLAB environment, where we used inbuilt functions for single-level 2D wavelet decomposition (dwt2) approximation coefficients matrix, rotation invariant local binary patterns (extractLBPFeatures) with 10×10 cells [48] and the linear multi-class support vector machine (fitcecoc).

**Figure 3:**
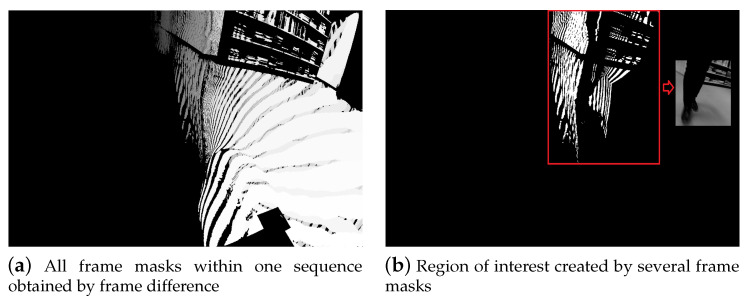
Movement detection and bounding box extraction.

**Figure 4:**
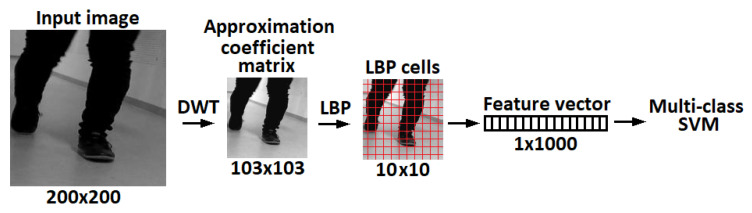
Feature extraction flow chart.

- *Setup:* To acquire the regions of interest (ROI) for the moving object in each frame (hand or foot), in this experiment, we applied simple and fast frame difference. If the difference is greater than 80 pixels for the foot and 30 pixels for the hand videos (these values were found empirically), then the resulted difference frame will be recorded as a binary mask. Figure 3a shows an example of all masks within one sequence, where the color transition from dark gray to white represents transition from the start of the video to the end of the video. For the foot sequences, the bounding box for a particular frame is created by taking that frame and then superimposing the previous 10 frames, and the next 10 frames that contain binary masks (identical to a sliding window). In other words, a binary image was formed by repeating the logical OR operation for 21 consecutive frames after which a bounding box has been found for the detected region as shown in Figure 3b. On the other hand, for the hand sequences, a fixed bounding box was created by using OR operation for all binary frames. After drawing the bounding box, the images were cropped and then resized to 200×200 pixels.
- *Experiments:* For the same subject, the total number of frames depend on the video length and therefore can not be fixed to a specific amount. The image features were extracted from an approximation coefficients matrix, which is one of the single-level two-dimensional wavelet decomposition method outputs. In particular, we used the Symlet wavelet to find results of the decomposition low-pass filter. Afterwards, the extracted rotation invariant local binary patterns from the output of wavelet decomposition were obtained as feature vectors to train a linear one-versus-one multi-class support vector machine. This process is shown in Figure 4. Finally, the fusion results obtained via majority voting, where a video label was determined by independent foot and hand frames. Results for single frame recognition and fusion can be found in Table 1. Note that, with a limited amount of data, taking into account that a random prediction classifier score in our problem of 77 labels is 1.3% accurate, which can be still considered as a reasonably good performance for the base line method.

#### 4.2.2. Dictionary Learning

Sparse based signal processing is a well-established method in the field of Computer Vision. This success is mainly due to the fact that important classes of signals such as audio and images have naturally sparse representations with respect to fixed bases (i.e., Fourier, wavelet), or concatenations of such bases [49]. It has been applied to many Computer Vision tasks such as face recognition [50], image classification [51], denoising [52], etc. In particular, the robust face recognition via sparse representation (SRC) algorithm proposed in [49] uses sparse representation for face recognition. In this method, the basic idea is to form an over-complete dictionary by using the training faces and then classifying a new face by searching the sparsest vector in this dictionary. Hence, this technique is called dictionary learning. Unlike conventional methods such as Eigenface and Fisherface, the dictionary learning can achieve superior results without any explicit feature extraction [53]. This superiority makes the SRC method a convenient method to employ in recognition tasks.

**Figure 5:**
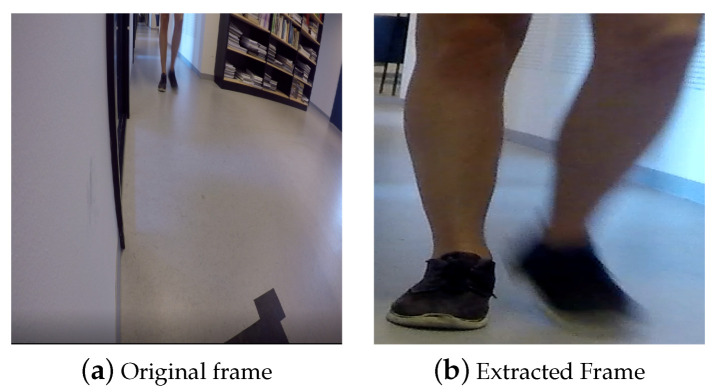
Sample of the extracted frame using a Kalman tracker.

- *Setup:* As a prior step to employ the SRC method for the classifier, we select the frames where both hand and feet are visible. For this, we used the Kalman visual tracker [54], which also defines the ROI within the associated selected images. Figure 5 shows examples of extracted frames to be used in the training stage of Dictionary Learning.
- *Experiments:* For the dictionary learning-based method, we employ the sparse representation classifier (SRC) of [49] for independent frame analysis. We randomly selected 50 extracted frames per subject in the training phase. Therefore, we generate a dictionary of size 100×3850 with a patch dimension (feature size) of 100 for each subject. This value of feature size has been found experimentally to provide the best performance. Then, at the test stage, using the same feature size, we attempt to recover the sparsest solution ($l_{1}$-minimization) to linear equation Ax=b, where A is the generated dictionary and y is a test image vector, respectively. The obtained results are shown in Table 2. As it can be seen, from both Table 1 and Table 2, conventional spatial appearance models provide poor classification results in all evaluated scenarios. This suggests that a more effective feature extractor is needed in this context.

### 4.3. Deep Learning

Deep Neural Networks, and especially Convolutional Neural Networks (CNN), have gained a lot of attention due to their state-of-the-art classification performance in many Computer Vision tasks since the breakthrough of AlexNet architecture [55] in the 2012 ILSVRC (ImageNet Large-Scale Visual Recognition Challenge).

**Figure 6:**
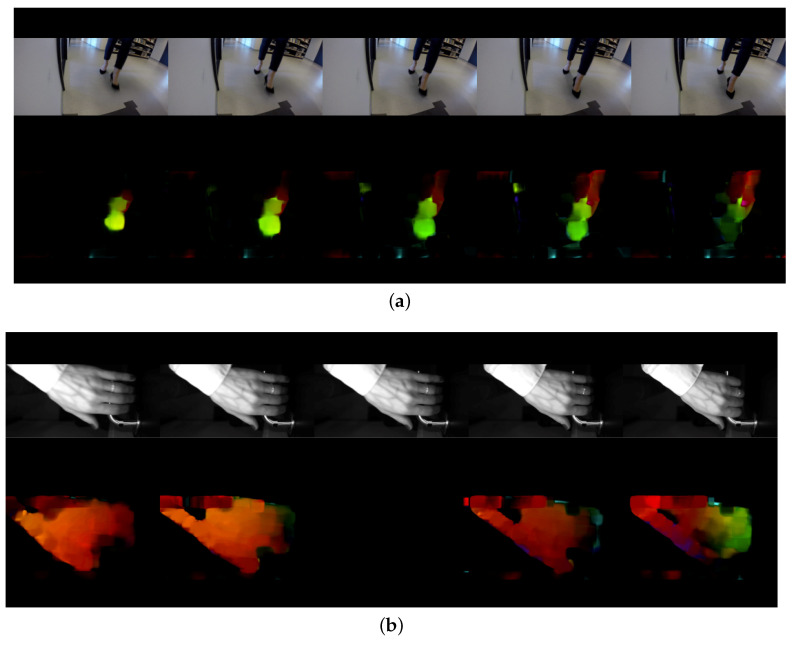
The heat map generated from an optical flow vector of consecutive video frames per each subject’s modality (**a**) and the corresponding heat map (**b**).

**Figure 7:**
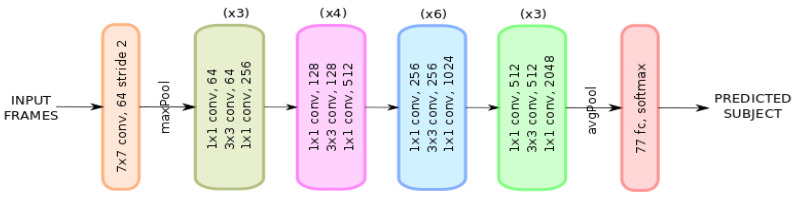
ResNet-50 neural network architecture [56].

In the context of this work, we opted to process the video frames as individual RGB images from both hand and feet datasets given the limited amount of data. However, for completeness, we have also considered motion maps as network inputs since some motion features may be unique to each subject regardless of their clothing. To that end, we have extracted the Optical Flow (OF) values (u,v) of each pair of consecutive video frames for each subject. The resulting OF values can then be used to generate a heat map that may potentially describe the motion features. Figure 6 shows a sample heat map generated by the Optical Flow vector of consecutive video frames of both hand and foot modality per subject. The rest of the simulations are arranged as follows:

- *Setup:* Since the dataset under study can be clearly linked to a classification problem, we have found it convenient to conduct our experiments on a standard ResNet-50 neural network architecture as shown in Figure 7. ResNet-50 has been proved to have a faster performance and lower computational cost compared to those of standard classification architectures such as VGG-16 due to its skip connection configuration [56]. For this purpose, we have constrained the input data (frames) to a [224×224] image size, batch size of 32 and output classes to 77 (number of eligible subjects) during the training phase. We left the number of input channels as a degree of freedom that will be set according to the different experiments we conducted.
- *Experiments:* In order to utilize the network, we have arranged the input to the network in the following four fashions:
  1. ***Appearance***—In this setting, the extracted frames, as before, are fed to the network for both the hand and foot datasets. Hence, input channel dimension is set to 3 due to the RGB nature of the frames. The recognition accuracy rate of this network is reported in Table 3.
  2. ***Optical Flow (OF)***—OF values (u,v) are extracted for each consecutive pair of frames for both the hand and foot in the dataset. In this case, we set the input channel parameter to 2 due to the OF dimensionality. Table 4 summarizes the results of this setting.
  3. ***Appearance + OF***—We apply an early fusion to the extracted frames and OF calculated values for both hand and feet datasets. That is, a 5-channel input parameter is set in order to match the RGB(3) + OF(2) new dimension. The results of this simulation are tabulated in Table 5.
  4. ***Appearance + Optical Flow (late fusion)***—We finally bring appearance analysis and OF analysis, computed separately, together. From each ‘branch’ output, we apply the same principle from the late fusion modality and study its performance. The result of this mode category can be seen in Table 6.

As it can be seen from the results in all of the tables, one can observe that the conventional techniques for both modalities and the late fusion can not effectively utilize the spatial information of the modalities and hence they may not be good candidate methods to be used in the context of a real solution for several reasons: on one hand, handcraft features can not properly model fine-grain information present in the data. Those small details are indeed the ones that may identify properly the subject in this challenging scenario. On the other hand, even for handcraft methods, the limited amount of data per subject in this dataset reduces the generalization capability of handcraft strategies. Still, please note that a random prediction guesser in this scenario will achieve 1.3% accuracy. Thus, an accuracy over 50% and a better result of the combined hand–foot model shows that handcraft methods, up to some degree, are able to learn some discriminative features and their complementary nature.

**Figure 8:**
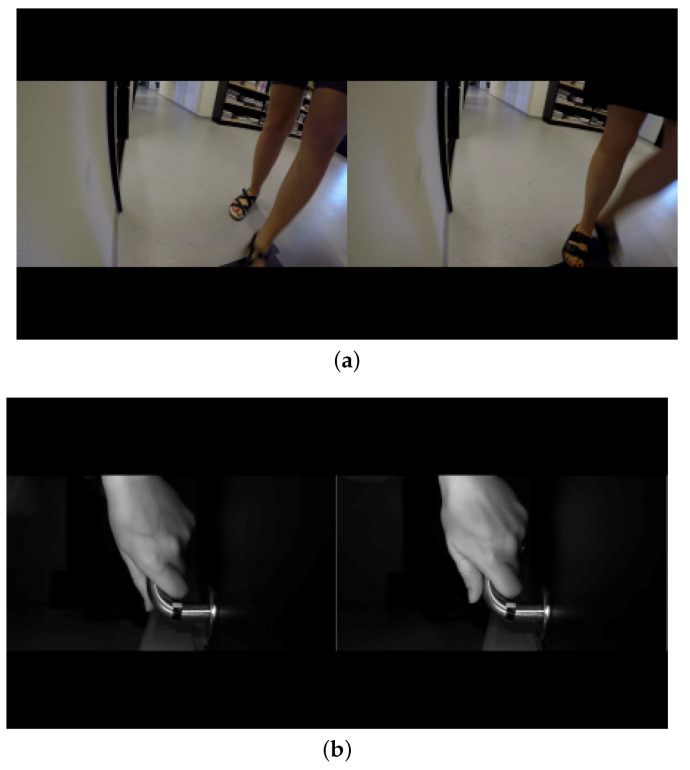
Appearance of misclassified examples. Left frames are being misclassified as belonging to the subject on the right. Misclassified hand modality of a subject (**a**). Misclassified foot modality of a subject (**b**).

In Deep Learning, however, we find the best setup classification results (84.4% accuracy) when analyzing the appearance per subject sequence modality—that is, when we use the whole sequence of frames per subject to determine the resulting class. This makes sense because some uncertain frame predictions do not normally contribute too much to the subject’s final estimation. We could imagine these misclassification outcomes as noisy samples, which are mostly cancelled out when averaging multiple data. Only the hand model achieves better performance than the only foot one. It was somehow expected because of the more controlled recording of hands and the freedom of the subject in terms of walking, i.e., different walking paths, different point of view, and different scales because of the distance to the camera. Interestingly, the late fusion combination increases around four points the results of the hand, suggesting that complementary and discriminative features are captured by the deep approaches. Some visual misclassified examples are shown in Figure 8. It can be seen that the frame on the left was misclassified as belonging to the same subject on the right. Some explainability can be found by just visual inspection (resemblance between subjects appearance). On the other hand, we find the worst performance when analyzing OF per independent frame analysis modality (32.6% and 35.1% accuracy). We believe motion can produce complementary features to the appearance ones and benefit from its appearance invariant descriptor. However, in order to obtain an increase in performance because of the use of motion, additional data and further strategies to mitigate the overfitting effect (e.g., data augmentation) should be considered.

## 5. Conclusions

In this paper, we presented a dataset containing hand and foot sequences for 77 subjects with the goal of performing automatic people recognition under privacy constraints. The dataset was collected using both RGB and near/infrared camera. We carried out extensive simulations using: (1) handcraft conventional techniques such as LBP, DTW, SRC, and SVM, and (2) deep learning. The results show that poor recognition performance is achieved when applying handcraft techniques, independently of the usage of hand or foot modality. On the other hand, the ResNet-50 deep model evaluated achieves a recognition rate of over 70% for feet and 80% for hands, further improved when fused, showing their complementary nature, and obtaining a final score of 84.4%. Interestingly, the inclusion of optical flow maps to enrich the appearance network channel did not show any improvement. This could have happened because of the limited amount of training data available per participant in the data set. All in all, spatial appearance deep learning showed a high generation performance to recognize users by the combination of hand and foot data. 

## Figures and Tables

**Table 1:** Recognition rate in (%) of the DWT-LBP-SVM approach.

Accuracy	
Modality	Independent frames DWT-LBP-SVM
Hand	37.78%
Foot	34.12%
*Hand+Foot* *(late fusion)*	57.14%

**Table 2:** Average recognition rate in (%) of the network for sparse representation classifier.

Accuracy (Dictionary Learning)	
Modality	Independent frames SRC
Hand	49.1%
Foot	41.3%
*Hand+Foot* *(late fusion)*	54.1%

**Table 3:** Average recognition rate in (%) of the ResNet network for the appearance model.

Accuracy (Deep Learning)			
No. of Samples	Modality	Independent Frames (appearance)	Subject Sequence (appearance)
2889	Hand	70.0%	80.5%
10195	Foot	58.6%	70.1%
	*Hand+Foot* *(late fusion)*	—	84.4%

**Table 4:** Average recognition rate in (%) of the ResNet network for the OF model.

Accuracy (Deep Learning)			
No. of Samples	Modality	Independent Frames (Optical Flow)	Subject Sequence (Optical Flow)
2812	Hand	32.6%	62.3%
10118	Foot	35.1%	48.1%
	*Hand+Foot* *(late fusion)*	—	59.7%

**Table 5:** Average recognition rate in (%) of the ResNet network for both the appearance and OF model.

Accuracy (Deep Learning)			
No. of Samples	Modality	Independent frames (appearance+ OF)	Subject Sequence (appearance+ OF)
2812	Hand	34.7%	62.3%
10118	Foot	54.8%	71.4%
	*Hand+Foot* *(late fusion)*	—	71.4%

**Table 6:** Average recognition rate in (%) of the ResNet network for the late-fused of both the appearance and OF model.

Accuracy (Deep Learning)	
Modality	Subject Sequence
	Appearance+OF/ Late Fusion
*Hand+Foot*	83.1%
